# Claw and limb disorders in 12 Norwegian beef-cow herds

**DOI:** 10.1186/1751-0147-49-24

**Published:** 2007-09-24

**Authors:** Terje Fjeldaas, Ola Nafstad, Bente Fredriksen, Grethe Ringdal, Åse M Sogstad

**Affiliations:** 1 Norwegian School of Veterinary Science, PO Box 8146 Dep. 0033 Oslo, Norway; 2 Animalia, Norwegian Meat Research Centre, PO Box 396 Økern, 0513 Oslo, Norway; 3 Department of Norwegian Cattle Health Services, TINE Norwegian Dairies, PO Box 58, 1431 Ås, Norway

## Abstract

**Background:**

The main aim of the study was to assess the prevalence of claw and limb disorders in Norwegian beef-cow herds.

**Methods:**

Twenty-six herds with ≥15 cow-years were selected by computerized systematic assignment from the three most beef cattle-dense regions of Norway. The study population consisted of 12 herds with 28 heifers and 334 cows. The animals were trimmed and examined once by claw trimmers during the late winter and spring of 2003. The seven claw trimmers had been taught diagnosing and recording of claw lesions. Environment, feeding and management routines, age and breed, culling and carcass characteristics were also recorded.

**Results:**

Lameness was recorded in 1.1% of the animals, and only in hind claws. Pericarpal swellings were recorded in one animal and peritarsal lesions in none. In total, claw and limb disorders including lameness were recorded in 29.6% of the animals, 4.1% with front and 28.2% with hind limb disorders, respectively. Most lesions were mild. Laminitis-related claw lesions were recorded in 18.0% of the animals and infectious lesions in 16.6%. The average claw length was 84 mm in front claws and 89 mm in hind claw. Both laminitis-related and infectious claw lesions were more prevalent with increasing age. Carcasses from animals with claw and limb disorders were on average 34 kg heavier than carcasses from animals without such disorders (p = 0.02). Our results also indicate association between some management factors and claw lesions.

**Conclusion:**

The study shows that the prevalence of lameness was low in 12 Norwegian beef-cow herds compared to beef-cattle herds in other countries and also that there were less claw and limb disorders in these herds compared to foreign dairy-cattle herds. The prevalence of lameness and white-line fissures was approximately the same as in Norwegian dairy herds whereas less dermatitis, heel-horn erosions, haemorrhages of the sole and the white line and sole ulcers were recorded.

## Background

Lameness is an important cause of reduced animal welfare and has been shown to cause substantial economical losses in dairy and beef-cattle herds [1,2]. Diseases of the feet account for ≈90% of all lameness cases in dairy cattle [3,4] and ≈70% in feedlots [5,6].

Claw disorders can be divided into three main categories according to their aetiology; infectious/partly infectious, metabolic/mechanical and traumatic [7]. Infectious and partly infectious claw lesions as dermatitis, heel-horn erosions and interdigital phlegmones are mainly influenced by the environment. Haemorrhages of the sole and the white line, sole ulcers and white-line fissures traditionally have been described as retrospective signs of subclinical laminitis, but lately by the term "claw-horn disruption" [8]. Important traumatic injuries are pedal bone fractures and traumas to the sole and interdigital space by foreign bodies. Laminitis (pododermatitis aseptic diffusa), laminitis-related lesions and injuries (bruises, lacerations and broken bones) are considered to be the most important non-infectious diseases in feedlot cattle [9]. To our knowledge, clinical prevalence of claw and limb disorders in beef-cow herds has not been reported before.

Environmental factors have huge influence on the incidence of claw lesions. *Stanek et al*. [10] found that the claw condition of fattening bulls was worse in a tie-stall system versus an outdoor untied paddock system, whereas *Lawrence et al*. [11] found that wet pen conditions increased both hoof growth and wear in Angus steers. Most studies have found that dairy cattle housed in free stalls have a higher claw lesion score than cattle in tie stalls [12-15]. The negative influence of confined dairy systems can be reduced by a well designed housing system [16].

In 2002 there were 48.497 beef cows in Norway versus 40.267 in 2000 [17]. The mean number of cow-years per herd was 15 and 14, respectively. Beef-cow production requires less labour, time and expenses compared to milk and meat production in dairy herds. Less meat from dairy herds and consumers demanding high quality steaks probably also results in further increase in beef-cow production in Norway. Herd sizes are expected to increase and more intensive production will probably predispose for more disease. In Norway, monitoring of health and disease is poorer in beef herds than in dairy herds. The recording system for production and diseases in beef-cow herds, the Norwegian Beef Cattle Herd Recording System (NBCHRS), which also includes claw and limb diseases, is established, but the health records are not complete.

The present study was part of a project on claw health of Norwegian cattle and the aim was to assess the prevalence of claw and limb disorders in Norwegian beef-cow herds. Some associations to breed, age, environment, management, reproduction and carcass characteristics are also assessed.

## Methods

### Selection procedure

We designed a cross-sectional study. Twenty-six herds registered in the NBCHRS and with ≥15 cow-years from the three most beef cattle-dense regions of Norway were sampled by computerized systematic assignment. Fifteen herdsmen accepted to take part in the study. Three herds were excluded because of non-compliance in connection with the trimming and recording of claw lesions. Claw and limb disorders and claw length and shape in cows and heifers ≥ 18 months of age were recorded.

### Study population

The final study population consisted of 12 herds: 6 in region I (Hedmark/Oppland), 3 in region II (Rogaland) and 3 in region III (Trøndelag). The total number of beef-cow herds with ≥ 15 cow-years within the three regions was 213, 71 and 169. Respectively 66, 14 and 45 of these herds were members of the NBCHRS. The total number of beef-cow herds with ≥ 15 cow-years in Norway was 1782 and 232 were members of the NBCHRS.

The mean number of cows and heifers in the study herds was 30 and the total number was 362; 28 heifers (more than 30 days from first calving) and 334 cows. Data from 337 of these animals were available from the NBCHRS. Housing, management, feeding and cow variables in each herd are in Table 1, 2 and 3.

**Table 1:** Housing systems and management factors in 12 Norwegian beef-cow herds (2003)

Herd	Region	n	Housing system	Floor	Walking area	Resting area	Feeding area	Exercise yard	Days at pasture (cow/heifer)	Routine claw trimming
A	I	15	Free stall	Other	Solid	Cubicles	Solid	Yes	66	No
B	I	45	Both	Concrete	Slatted	Cubicles	Slatted	Yes	118	No
C	I	31	Free stall	Concrete	Slatted	Slatted	Slatted	No	165	No
D	I	35	Free stall	Other	Deep litter	Deep litter	Solid	Yes	82/112	Yes
E	I	21	Free stall	Other	Deep litter	Deep litter	Deep litter	Yes	77/213	No
F	I	19	Free stall	Concrete	Slatted	Slatted*	Slatted	No	132/127	No
G	II	16	Tie stall	Concrete				No	153	No
H	II	33	Free stall	Other	Deep litter	Deep litter	Solid	Yes	364	No
I	II	18	Free stall	Concrete	Slatted	Cubicles	Slatted	No	364/144	No
J	III	33	Tie stall	Concrete				No	117/132	No
K	III	17	Free stall	Concrete	Slatted	Cubicles	Slatted	No	200/91	No
L	III	79	Free stall	Concrete	Solid	Cubicles	Solid	No	109	No

* Mats of rubber used around calving

**Table 2:** Feeding and feeding routines in 12 Norwegian beef-cow herds (2003)

Herd	Region	n	Types of roughage feed	Concentrate heifers	Concentrate before calving	Concentrate after calving	Mineral or vitamin additives	Salt lick with minerals
A	I	15	Round bale grass silage, straw	Yes	No	No	Yes	Yes
B	I	45	Round bale grass silage, ammonia-treated straw	Yes	No	No	Yes	Yes
C	I	31	Grass silage, ammonia-treated straw, hay, round bale grass silage	Yes	Yes	Yes	No	Yes
D	I	35	Round bale grass silage	Yes	Yes	Yes	Yes	Yes
E	I	21	Round bale grass silage, ammonia-treated straw	Yes	Yes	No	Yes	Yes
F	I	19	Round bale grass silage	Yes	No	Yes	Yes	Yes
G	II	16	Grass silage, round bale grass silage	Yes	No	No	Yes	Yes
H	II	33	Grass silage, round bale grass silage	Yes	No	No	Yes	Yes
I	II	18	Grass silage	Yes	Yes	Yes	Yes	Yes
J	III	33	Round bale grass silage, ammonia-treated straw	Yes	Yes	Yes	Yes	Yes
K	III	17	Round bale grass silage, ammonia-treated straw, other	Yes	No	Yes	Yes	Yes
L	III	79	Round bale grass silage, ammonia-treated straw	Yes	Yes	Yes	No	Yes

**Table 3:** Claw trimming and cow variables of the herd at the time of trimming in 12 Norwegian beef-cow herds (2003)

Herd	Region	Cows (n)	Claw trimmer (id)	Season for trimming	Median date for calving	Mean age	Breed*
A	I	15	a	June	06/04	5.6	Ch/CU
B	I	45	a	June	17/02	3.9	He
C	I	31	a	May	14/05	6.1	CU/AA/He
D	I	35	a	May	22/03	4.8	He/CU/Li
E	I	21	b	April	12/05	4.8	CU/HE
F	I	19	b	January	06/03	5.8	CU/AA/Ch
G	II	16	c	March	01/04	4.8	Li/CU/He/Ch
H	II	33	c	February	-	6.0	CU/Li/AA
I	II	18	c	January	16/04	4.3	Si/CU
J	III	33	d/e	February	02/06	3.5	CU/Ch
K	III	17	f/g	January-February	05/03	4.7	Ch/CU
L	III	79	f/g	January-February	14/04	5.0	Ch/CU

*He = Hereford, Ch = Charolais, AA = Aberdeen Angus, Li = Limousine, Si = Simmental, CU = Crossing or unknown. The most numerous breed in each herd is the first written.

The Hereford breed was present in 5 of the herds and Aberdeen Angus, Charolais, Limousine and Simmental in respectively 3, 6, 3 and 1 of the herds. In total 72 Herefords, 97 Charolais, 14 Aberdeen Angus, 20 Limousines, 15 Simmentals, 93 cross-breds and 26 animals of unknown breed were included in the study. Animals with more than 75% of one breed were counted as pure-bred.

### Recording of data

All seven professional claw trimmers attended two courses covering observation and recording of lameness, claw trimming procedures, diagnosis, recording and treatment of claw lesions. Individual training was given to each claw trimmer at the initiation of the practical work. For practical reasons two claw trimmers cooperated in some of the herds. All trimmers had previously participated in a study of claw health in dairy cattle [15].

The cows were trimmed and examined once during the period from the 15th of January 2003 to pasture season started. The last herd was visited on the 12th of June. Lameness was assessed when the animal was moved to the trimming chute as absent (no notation), moderate (1) or severe (2) (Table 3). Pericarpal and peritarsal swellings and wounds were recorded as absent (no notation), swelling (1), wound (2) or both swelling and wound (3). Claw shapes were recorded as normal (no notation), asymmetric (1) or corkscrewed (2). Claw lesions were diagnosed on the basis of macroscopic examination before and after trimming to the correct claw shape. The trimming technique included levelling the two claws, aiming for symmetric bulbs. The axial and abaxial walls were both intended to be parts of the bearing surface and the two claws were trimmed flat and balanced with each other. The caudal 2/3 of the axial sole of both claws was dished out. Dermatitis, heel-horn erosions, haemorrhages of the white line and the sole, sole ulcers and white-line fissures were scored as absent (no notation), mild (1), moderate (2) or severe (3). Definitions in Table 4 are adapted from *Bergsten *[18]. We used only one variable for "dermatitis" because the occurrence of digital dermatitis is close to zero in Norway and we assumed that the recorded cases would be interdigital dermatitis. The presence of double sole, interdigital hyperplasia, horizontal and vertical fissures, interdigital phlegmon and papillomatous dermatitis was also recorded. The recording protocol did not differentiate between the inner and the outer claw because most lesions occur in the outer hind claw and the inner front claw [19,4]. The term "claw and limb disorders" includes lameness, pericarpal and peritarsal swellings or wounds and all claw lesions in this study, but not asymmetric or corkscrewed claws.

**Table 4:** Definition of lameness and claw lesions recorded at trimming

Lesion	Score*	Definition
Lameness	1	Asymmetric gait, bearing weight on all limbs
	2	Avoiding weight-bearing on one or more limbs
Dermatitis	1	Superficial, hyperaemic, slightly Exudative lesion of the digital/interdigital skin
	2	Exudative, slightly ulcerative lesion with thickening of the skin
	3	Ulcerative, spontaneously bleeding lesion with thickening of the skin and great pain
Heel-horn erosion	1	Slight defects of the horn integrity, pits and small fissures
	2	V-shaped fissures or craters of the heel/bulb not affecting corium
	3	V-shaped profound fissures or craters affecting corium of the heel/bulb
Haemorrhages of the white line	1	Slight haemorrhagic discoloration
	2	Moderate haemorrhage on a single spot or several superficial haemorrhages covering > 20% of the white line
	3	Profound haemorrhage on a single spot or extensive haemorrhagic discoloration covering > 50% of the white line
Haemorrhages of the sole	1	Slight haemorrhagic discoloration
	2	Moderate haemorrhage on a single spot or several superficial haemorrhages covering > 20% of the sole surface
	3	Profound haemorrhage on a single spot or extensive haemorrhagic discoloration covering > 50% of the sole
Sole ulcer	1	Exposed, unaffected corium
	2	Granulation tissue, necrosis, purulent exudates and separation of the sole horn
	3	As score 2 with additional affection of the deeper structures of the claw
White-line fissure	1	Fissure, which disappear with deep cut beneath normal trimming level
	2	Deep fissure perforating next to the corium of sole or wall
	3	Corium is affected with purulent exudates, eventually with necrosis, granulation tissue and separation of the wall and/or sole

* Lameness: score 1 = moderate, score 2 = severe. Claw lesions: score 1 = mild, score 2 = moderate, score 3 = severe. Absence of lameness or claw lesions: no notation

Identity, age, date for calving, breed and events as disease and carcass characteristics were extracted from the NBCHRS. When associations between age and claw lesions were assessed, the animals were separated in young (2–4 years), medium-aged (5–7 years) and old animals (8–10 years). Conformation class and fat cover class were defined according to the EUROP grading system as defined by the EU [20,21]. Data on housing system, environment, feeding and management were collected by visits and questionnaire survey by one animal husbandry adviser.

### Statistical analyses

The statistical analyses were performed in the SAS-PC System^® ^Version 9.1 for Windows at cow and herd level. PROC UNIVARIATE, PROC MEANS and PROC FREQ were used for the descriptive analyses.

Two different unconditional logistic regression models were performed using the presence or absence of infectious and laminitis-related claw lesions as dependent variable, and age group (young = 2–4 years, medium = 5–7 years, old = ≥8 years) as independent variable (PROC LOGISTIC).

For animals that had been slaughtered within two years after claw inspection, general linear models (GLM) with carcass weight, conformation class and fat cover class as dependent variables were performed with "disorder" (presence of any claw or limb disorder, yes/no) as independent variable and breed as adjusting variable. The fit of the models was assessed by the R^2^-values.

At herd level, univariate statistics were generated for all environmental factors in relation to the occurrence of 1) claw and limb disorders, 2) laminitis-related claw lesions and 3) infectious claw lesions. For laminitis-related claw lesions, several of the environmental factors were statistical significant. All these factors were included as independent variables (fixed effects) in a preliminary multivariate (GLM) model with the herd prevalence of laminitis-related claw lesions as dependent variables. Two-tailed tests were applied. The type III F-test was used as elimination criterion. The modelling was manually conducted by stepwise backward elimination of variables one by one, using a p-value of 0.05 as the level for exclusion from the model. The least square means were estimated for all levels of significant independent variables in the final model.

General linear model (GLM) with calving interval as dependent variable was performed using PROC GLM, with claw and limb disorders (yes/no) as independent variables and breed as adjusting variable.

## Results

### Prevalence of claw and limb disorders

Lameness was recorded in 1.1% of the animals, and only in hind limbs (Table 5). Pericarpal swellings were recorded in one animal. In total, claw and limb disorders were recorded in 29.6% of the animals, 4.1% with front and 28.2% with hind limb disorders, respectively.

**Table 5:** Prevalence of lameness, pericarpal and peritarsal swellings, asymmetric claws, corkscrewed claws and claw lesions on individual and herd level recorded at claw trimming of 362 animals in 12 Norwegian beef-cow herds (2003)

Disorder	Individual prevalence (%)		Herd prevalence*	
	Front claws Mean (SD)	Hind claws Mean (SD)	Min	Max
Lameness	0 (0)	1.1 (10.5)	0	5.1
Pericarpal and peritarsal swellings	0.3 (5.3)	0 (0)	0	1.3
Asymmetric claws	4.4 (20.6)	6.9 (25.4)	0	47.4
Corkscrewed claws	0	4.2 (20.0)	0	26.7
Infectious claw lesions	1.7 (12.8)	16.3 (37.0)	0	58.2
Dermatitis	0 (0)	2.2 (14.7)	0	7.6
Heel-horn erosions	1.7 (12.8)	16.1 (36.8)	0	57
Laminitis-related claw lesions	2.2 (14.7)	16.9 (37.5)	0	60.6
Haemorrhages of the white line	0.8 (9.1)	3.0 (17.2)	0	18.2
Haemorrhages of the sole	0.6 (7.4)	7.8 (26.8)	0	25.0
Sole ulcers	0.3 (5.3)	1.4 (11.7)	0	6.7
White-line fissures	0.8 (9.1)	8.3 (27.6)	0	36.4
Double sole	0	1.4 (11.7)	0	12.5

*Herd prevalence is calculated as percentage of cows with lesions

Most claw lesions in front and hind claws were score 1. All together 20.6% of the lesions were score 2: Heel-horn erosions (20 out of 64 cases); sole ulcers (3 out of 6 cases) and dermatitis (3 out of 8 cases). Only one score 3 (heel-horn erosions) was recorded.

Infectious claw lesions including dermatitis and heel-horn erosions were recorded in 16.6% of the animals, varying from 0–58.2% on herd level, and laminitis-related claw lesions including haemorrhages in the white line and the sole, sole ulcers, white-line fissures and double soles were recorded in 18.0% of the animals, varying from 0–60.6% on herd level (Table 5 and 6). Infectious lesions were 9.8 times more frequent in hind claws than in front claws, whereas laminitis-related lesions were 7.6 times more frequent in hind claws. Laminitis-related claw lesions were recorded in 11 out of the 12 herds (92%), whereas infectious claw lesions were recorded in 4 out of 12 herds (33%). Vertical fissure and interdigital hyperplasia (corns) were both only recorded in one animal. Interdigital phlegmon, horizontal fissure and papillomatous dermatitis were not recorded in any animal.

**Table 6:** Prevalence of laminitis-related and infectious claw lesions in each of 12 Norwegian beef-cow herds (2003)

Herd	n	Laminitis-related claw lesions (%)	Infectious claw lesions (%)
A	15	6.7	0
B	45	4.4	0
C	31	0	0
D	35	11.4	2.9
E	21	14.3	0
F	19	26.3	42.1
G	16	31.3	0
H	33	60.6	15.2
I	18	33.3	0
J	33	3	0
K	17	0	0
L	79	20.3	58.2

### Claw length and shape

The average front-claw length was 84 mm, varying from 61–100. Hind claws were 89 mm (67–102). Recordings of asymmetric and corkscrewed claws are in Table 4. Corkscrewed claws were mainly recorded in Charolais (10%) and Limousine (15%). Asymmetric claws were mainly registered in Aberdeen Angus (23%), Limousine (15%) and Charolais (14%).

### Effect of age and breed on claw lesions

**Figure 1:**
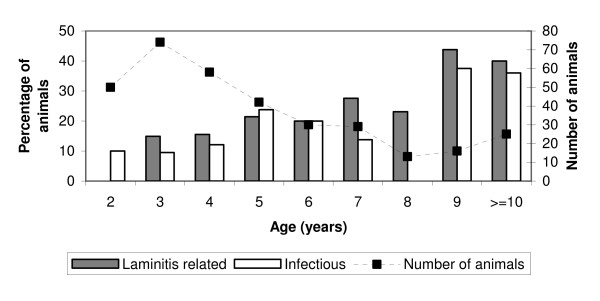
Total prevalence of laminitis-related and infectious claw lesions related to age in 12 Norwegian beef-cow herds.

**Figure 2:**
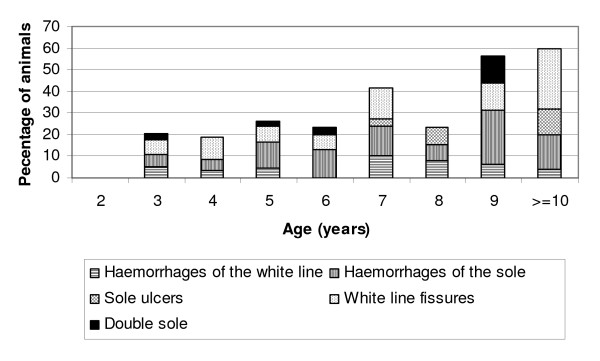
Laminitis-related claw lesions related to age in 12 Norwegian beef-cow herds.

**Figure 3:**
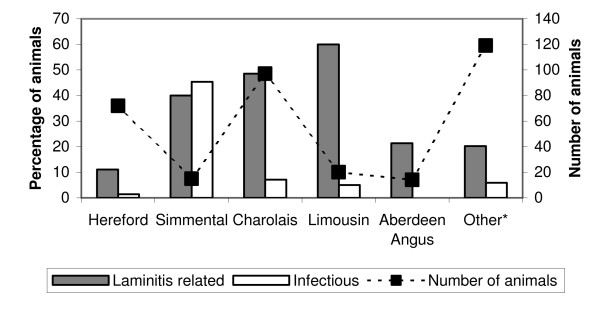
Total prevalence of laminitis-related and infectious claw lesions related to breed in 12 Norwegian beef-cow herds.

The distributions of laminitis-related and infectious claw lesions related to age are in Figure 1 and the distribution of different laminitis-related lesions related to age is in Figure 2. There were more laminitis-related lesions in medium-aged (OR = 2.4, 95% CI 1.2–4.6) and old animals (OR = 4.8, 95% CI 2.3–9.8) compared to young animals (p < 0.0001). Infectious lesions were also more prevalent in older cows, and the corresponding numbers were 2.1 (1.1–4.2) and 3.3 (1.5–7.1) (p < 0.01). The distributions of laminitis-related and infectious claw lesions related to breed are in Figure 3.

### Claw and limb disorders related to reproduction and carcass characteristics

The average calving interval was 366 days for cows with disorders, and 355 days for cows without, but the difference was not significant when adjusted for herd. The percentages of animals that were slaughtered one and two years after claw trimming were not significantly different between cows with and without disorders. Within the group of animals that were slaughtered within two years after claw trimming, the animals with disorders had on average a higher carcass conformation class (5.9) than the animals without disorders (4.8). The difference was, however, not significant after adjusting for breed. Carcasses from animals with disorders were on average 34 kg heavier than carcasses from animals without disorders, when adjusting for breed (p = 0.02), but had similar fat cover class.

### Claw lesions related to environmental factors and management

None of the herd-level environmental factors was associated with the herd prevalence of claw and limb disorders. For laminitis-related lesions, several factors were significant when looked at separately (Table 7). However, only "region" remained in the multivariate model. For infectious claw lesions, the only significant factor was frequency of change and supplementation of litter in calf pens, with average prevalences of 0, 9 and 58% for "daily", "when needed" and "weekly", respectively (p < 0.01, R^2 ^= 0.65).

**Table 7:** Univariate statistics for environmental factors and management that were associated with herd prevalence of laminitis-related lesions in 12 Norwegian beef-cow herds (2003)

Factor	β	Value	Number of herds	Mean prevalence	p-value	R^2^
Presence of isolated room for staff		Yes	6	0.08	0.03	0.38
		No	6	0.30		
Hot and cold water available		Yes	8	0.12	0.05	0.33
		No	4	0.34		
Region		I	6	0.12	<0.01	0.75
		II	3	0.46		
		III	3	0.08		
Days at pasture – cows	0.001				0.02	0.42
Days at pasture – heifers	0.002				<0.01	0.52

## Discussion

### Representativity and general discussion

Five months is a relatively long recording period for a cross-sectional study and might have biased our results. However, because of considerable climate differences between the three regions it was impossible to avoid trimming in different months with this study design. It was important for us to include three regions to achieve the largest possible study population.

Average herd sizes in our study were larger than the Norwegian average, but are probably representative for future beef-cow herd sizes in Norway. The study was part of a project where the main aim was to study claw health in dairy herds, and systematic errors caused by differences in claw-trimming practices and diagnosing of claw lesions are discussed by *Sogstad et al*. [15]. All claw trimmers in our study also participated in the study of dairy herds where a lack of cluster effect within claw trimmer (except for heel-horn erosions in front and hind claws and white-line fissures in front claws) indicated that agreement between trimmers was satisfactory. The recording protocol in this study was identical to the protocol in the dairy-herd study and results from our beef and dairy-cattle studies are compared below. *Manske *[22] found differences in recording among claw trimmers, and underreporting of mild and common lesions were marked. Underreporting might have biased our results, but we expect the most-important lesions to be recorded. There is probably an underestimation of lameness in the present study. It was difficult to observe lameness when the cows were moved to the trimming chute. Consequently the sensitivity for detection of lameness was low and the recorded prevalence should be considered as an assessment of moderate or rather severe lameness.

Herdsmen, who did not want to participate in the study, might have been less interested in maintaining good claw health. Consequently, bad claw health in their herds might have led to underestimation of the prevalence. On the other hand, bad claw health might have been an incentive for participation. Because of many drop-outs and exclusions the study population is small and cows are also separated on breeds. Nevertheless, prevalence of claw and limb disorders in beef-cow herds has hardly been presented before and if interpreted with care, the results should be useful.

### Lameness

The 1.1% prevalence of hind-limb lameness in this study was approximately the same as in our dairy-cattle study where hind-limb lameness was recorded in 1.2% of the animals [23]. In a Swedish study of dairy cows the prevalence of lameness was 5.1% [24]. *Roeber et al*. [25] found that the incidence of lameness of cattle was 26.6% for beef cows and 30.2% for dairy cows. Arthritic stifle joints were one of the most important causes of lameness in both beef and dairy herds. He also found that economic losses had increased since 1994 [2]). *Hird et al*. [26] reported that the highest costs of veterinary services in 57 Californian beef herds were related to dystocia, lameness and ocular carcinoma. Foot rot was the costliest disease causing lameness. *Stokka *[9] claimed that traumatic injuries are important causes of feedlot morbidity and mortality that often is not recognized until the animal has deteriorated substantially.

The low prevalence of lameness in our study might partly be explained by no foot rot, no infectious arthritis and no traumatic injuries. Compared to most other countries beef-cow herds in Norway are small and this might imply management which has positive influence on animal health. Foot rot and traumatic injuries also occur occasionally in Norwegian beef-cow herds, but our experience from practice and the present study indicate that the problem is small compared to in feedlot cattle where these disorders have a major influence on claws and limbs [27]. On the other hand, our material was small and such diseases might easily have been missed in the prevalence study.

### Infectious claw lesions

The low prevalence of dermatitis in this study might partly be explained by the fact that digital dermatitis is not established in Norway [28]. The prevalence of dermatitis was also lower than in the dairy-herd study [15] which shows that even interdigital dermatitis was not a problem in these beef-cow herds. The prevalence of heel-horn erosions was low compared to Norwegian free-stall dairy herds (39.6%), but more lesions were score 2.

Dermatitis and heel-horn erosion are infectious in origin and moisture and dirt are considered to be important predisposing factors. Manure has detrimental effects on horn [29]. Dairy cows in free stalls are at increased risk of getting dermatitis and heel-horn erosion relative to tied cows [24,30,15]. *Thysen *[13] found more heel-horn erosions both in free-stall dairy herds with slatted and full concrete alleys than in tie-stall herds, and more so when free stalls included concrete alleys. Most of the present herds were housed in free stalls, and the results indicate that interdigital dermatitis and heel-horn erosions are not widespread in Norwegian beef-cow herds. The results might partly be explained by low-intensity feeding, which usually results in drier manure, and a longer pasture period than in dairy herds. In agreement with *Somers et al*. [31] who found that restricted grazing time was associated with increased odds of interdigital dermatitis and heel-horn erosion, it is our experience that these lesions usually are in-door diseases. More heel-erosions in hind claws than in front claws also agree with *Sogstad et al*. [15]. This is probably explained by hind claws being more exposed to manure than front claws in any housing system.

### Laminitis-related claw lesions

The prevalence of recorded laminitis-related lesions including white-line and sole haemorrhages, sole ulcers, white-line fissures and double soles was low compared to Norwegian dairy herds. These lesions have a multifactorial aetiology and are influenced by nutrition, feeding routines, hormones around calving and external and internal mechanical forces [7]. According to *Stokka *[9] laminitis may be the number-one cause of foot problems both in dairy and feedlot cattle. However, when comparing with feedlots, it must be kept in mind that most of those herds consist of bulls on high-energy feeding whereas our herds were cows fed much roughage and small amounts of concentrates. More laminitis-related lesions in hind claws than in front claws in our study are in agreement with studies of dairy cattle [4,24,15]. Differences in claw shape, limb conformation, movement and shifting of weight make the hind claws more disposed [19], and hind claws are also more exposed to dirty environment.

The prevalence of haemorrhages of the white line and the sole was low compared to many studies of dairy cattle [32,33,30]*. Sogstad et al*. [15] found that 13.6% of hind feet in free-stall herds were affected by haemorrhages of the white line and 20% by haemorrhages of the sole. Lower prevalence of haemorrhages in beef-cow herds might be the result of low-intensity feeding. *Greenough et al*. [34] found that high-energy feed increased the prevalence of toe and heel haemorrhages in feedlot calves and heel haemorrhages in feedlot yearlings. External mechanical forces is also considered to cause claw-horn disruption and haemorrhages [16,35], but our material is too small and there is too much variation in housing and conformation systems for any conclusions on the influence of environment.

The prevalence of sole ulcers was also low compared to what has been found in most studies of dairy cattle [36,24] but approximately the same as in the Norwegian dairy cattle study. Sole ulcers are the result of haemorrhages and contusions in the corium leading to claw-horn disruption and possible infection [8]*. Thysen *[13] found that the prevalence of sole ulcers observed at claw trimming was not affected by the housing system, which is in agreement with *Sogstad et al*. [15]. This suggests that metabolic and hormonal factors are important in the pathogenesis of sole ulcers both in beef and dairy cattle.

White-line fissures being the most frequent laminitis-related lesion in our study partly agrees with *Smith & Brodersen *[37] who found that separation of the wall from the sole at the white line was the most frequent external lesion in lame feedlot cattle. *Mülling *[38] considered separation of the white line to be the result of weakening of the suspensory apparatus of the claw, haemorrhages, accumulation of exudates and impaired horn production, which again predisposes to infection. He also suggests that haemorrhages of the white line predispose to white-line fissures, but also that fissures might be caused directly by mechanical influences in the environment. The Norwegian dairy cattle study also indicated that direct mechanical influences including uneven forces from slatted concrete floors, is important for the development of white-line fissures [35]. Bad slats, narrow passageways, uncomfortable cubicles, overcrowding, increased competition and "bulling" activity have been suggested as negative factors in dairy free-stall herds [39,40]. Low prevalence of haemorrhages but relative high prevalence of white-line fissures in our study might indicate that direct mechanical influence is an important cause of the fissures. Some of these beef-cow herds were housed on slatted concrete floors while others were housed on solid concrete or deep litter, but the material was again too small to reveal associations between fissures and type of alley. *Smith & Brodersen *[37] found that there was a strong association between separation of the wall from the sole at the white line and internal claw lesions like osteomyelitis. The prevalence of white-line fissures was 36.4% in one herd in our study, indicating that this lesion can have serious consequences for meat production and animal welfare in some herds.

The relative high prevalence of double soles in one herd confirms that laminitis is a problem in some Norwegian beef-cow herds. Only one animal recorded with a vertical fissure is in contrast to cross-sectional surveys reported by *Clark et al*. [41] where the prevalence of vertical fissures in slaughtered beef cows in Western Canada was approximately 20%. Large lateral front claws were most prone to vertical fissures. The difference to *Clark et al*. [41] might partly be the result of vertical fissures being easier to diagnose at slaughter than at the farm. However, our result agrees with experience from practice that vertical fissure is seldom seen in Norwegian beef cows.

### Claw length and shape

Hind claws were on average longer than claws from Norwegian dairy cattle in free-stall housing [42]. Claws were also approximately 10 mm longer than claws in dairy cows housed in tie stalls with concrete flooring. This might be explained by three of the herds being housed with deep litter both in the resting and walking area, and that routine trimming was performed in only one of the herds. There might also be a real difference in claw size between Norwegian Red breed and beef-cow breeds. The prevalence of corkscrewed claws was on average approximately the same as in Norwegian dairy herds, but 26% of the animals being affected in one herd indicate that corkscrewed claws might cause problems in some herds because secondary lesions like haemorrhages of the sole, pedal ostitis and arthrosis of the distal interdigital joint are frequent in corkscrew claws [43,44]. The breed distribution of asymmetric and corkscrewed claws should be interpreted with care because of the low number of animals.

### Effect of age and breed on claw lesions

Both laminitis-related and infectious claw lesions were more prevalent with increasing age. This is partly in agreement with several studies which found more lameness with increasing age [45-47,24,35]. This might be the result of repeated scarring of the corium with irreversible and cumulative damage to claw tissue [8]. More haemorrhages of the sole with increasing age are in contrast to many studies of dairy cattle which found highest odds for haemorrhages of the sole in primiparous cows [32,24,35]. Dairy heifers are experiencing major changes in housing conditions, social environment, nutrition and physiologic demands which might lead to increased prevalence of haemorrhages in first lactation. Because beef-cattle heifers do not experience such dramatic changes around calving, fewer laminitic lesions including haemorrhages of the sole can be expected. *Stanek et al*. [10] found that claw condition grew worse with an increase in body weight, and higher body weight with increasing age might partly explain the relation between more claw lesions and increasing age. *Townsend et al*. [48] found that lameness in 326 young beef bulls was associated with weight. They predicted that the odds of lameness in the animal with the heaviest initial test weight was approximately seven times greater than in the animal with the lightest initial test weight. Foot rot, laminitis and minor traumatic injuries were evaluated to be the most important causes of lameness in their study.

*Townsend et al*. [48] found relation between lameness and breed and explained this by differences in claw shape, size, conformation and horn composition of the different beef-cattle breeds. Small groups in our study make comparison of different breeds difficult. There were 72 animals of the Hereford breed and 97 Charolais. The prevalence of laminitis-related lesions was 11% and 49%, respectively. The herd effect might be responsible for most of the difference, but the prevalence for the Charolais breed is still rather high. For infectious claw lesions, Simmental had the highest prevalence with 45%. However, this number refers to only 15 animals from one herd, and should be interpreted with care. Almost all dairy cattle in Norway are Norwegian Red, and the effect of breed must be kept in mind when these beef-cow herds are compared to the dairy-cattle herds.

### Claw and limb disorders related to reproduction and carcass characteristics

Longer calving interval in animals with claw and limb disorders versus animals without is in agreement with *Sogstad et al*. [23] who found associations between moderate and severe heel-horn erosions and sole ulcers and increased calving interval in dairy cattle. Cows with tender feet are more reluctant to walk, show less estrual activity and likely eat less than other cows. *Barth & Waldner *[49] found that lameness reduced the probability of satisfactory reproductive-soundness classification of beef bulls.

Higher conformation class and increased carcass weight in animals with disorders versus those without can probably be explained by the breed differences. An unanswered question is why more cows of heavy than light breeds were slaughtered within two years of the claw inspection. *Sogstad et al*. [50] found that lameness and lesions at the tarsus in dairy cattle were associated with lower conformation class and lower carcass weight, whereas sole ulcers were associated with higher conformation class. Our results might be a direct consequence of more claw and limb disorders in heavy breeds. However, the results might also be influenced by most lesions being mild.

### Claw lesions related to environmental factors and management

The small power in the study makes association between claw lesions and environment and management hard to detect. The relation between increased time at pasture for both cows and heifers and laminitis-related claw lesions was not expected because pasture usually is positive for claw health. However, if the animals are at pasture in winter, as they were in some herds, and the soil gets frozen, this might lead to increased external pressure on the claws. Muddy and frozen feed might also cause digestive disorders. The associations between the presence of isolated room for staff and available hot and cold water and laminitis-related lesions might indicate that management and attention indirectly influence claw health. "Region" probably remained in the model because some variables influencing on laminitis-related lesions were different in the 3 regions (Table 3). The distribution of both season for trimming and breed were skewed. It is not obvious how the recorded date for calving and age influenced our result. However, the herd with the highest prevalence of laminitis-related lesions, which was located in region II, also had a high mean age. Unfortunately the median date for calving in this herd was not reported and there might also be other unknown factors predisposing for laminitis-related lesions in this herd. Even though there was a lack of cluster effect within claw trimmer in our dairy-herd study we cannot exclude the possibility that different trimmers recording claw lesion might have biased the present result.

The variable "frequency of change and supplementation of litter in calf pens" is an indicator for the general hygienic level in the herds. Frequent change and supplementation of litter might have a direct preventive effect on infectious claw lesions, however, the investigated herd groups, cows and heifers, did not use these pens at the time of recording.

## Conclusion

Our study shows that the prevalence of lameness was low in 12 Norwegian beef-cow herds compared to beef-cattle herds in other countries and also that there were less claw and limb disorders in these herds compared to foreign dairy-cattle herds. Most claw lesions were mild, and the prevalence of lameness did not differ much from Norwegian dairy herds. Laminitis-related lesions were recorded in 18.0% and infectious claw lesions in 16.6% of the animals. White-line fissure was the most frequent laminitis-related lesion and heel-horn erosion the most frequent infectious lesion. Both laminitis-related and infectious claw lesions increased with age.

## Competing interests

The author(s) declare that they have no competing interests.

## Authors' contributions

TF contributed to the design of the study. He taught the claw trimmers correct trimming and how to diagnose and record claw lesions. He also made the draft of the manuscript. ON who knows the beef-cow production very well, contributed to the design of the study and was the main coordinator. He also helped drafting the manuscript. BF performed the statistical and epidemiological analyses and also wrote the main part of the chapter "Material and methods". GR visited the herds and recorded data on housing system, environment, feeding and management. ÅMS contributed to the design of the study and performed proof-reading of the recorded data. All authors read the manuscript several times and approved the final manuscript.

## References

[B1] Enting H, Kooij D, Dijkhuizen AA, Huirne RBM, Noordhuizen-Stassen EN (1997). Economic losses due to clinical lameness in dairy cattle. Livest Prod Sci.

[B2] Roeber DL, Mies PD, Smith CD, Belk KE, Field TG, Tatum JD, Scanga JA, Smith GC (2001). National market cow and bull beef quality audit-1999: a survey of producer-related defects in market cows and bulls. J Anim Sci.

[B3] Logue DN, Offer J, Kempson SA (1993). Lameness in dairy cattle. Ir Vet J.

[B4] Murray RD, Downham DY, Clarkson MJ, Faull WB, Hughes JW, Manson FJ, Meritt JB, Russel WB, Sutherst JE, Ward WR (1996). Epidemiology of lameness in dairy cattle: description and analysis of foot lesions. Vet Rec.

[B5] Griffin D, Perino L, Hudson D (1993). Feedlot lameness. University of Nebraska Extension Publication G93-1159-A.

[B6] Miskimins D, Shearer JK (2002). Predominant causes of lameness in feedlot and stocker cattle. Proceedings of the 12th International Symposium on Lameness in Ruminants: Florida, 9–13 January 2002; Orlando.

[B7] Greenough PR, Weaver AD, Eds (1997). Lameness in cattle.

[B8] Lischer CJ, Ossent P, Shearer JK (2002). Pathogenesis of sole lesions attributed to laminitis in cattle. Proceedings of the 12th International Symposium on lameness in Ruminants: 9–13 January 2002; Orlando.

[B9] Stokka GL, Lechtenberg K, Edwards T, MacGregor S, Voss K, Griffin D, Grotelueschen DM, Smith RA, Perino LJ (2001). Lameness in feedlot cattle. Vet Clin North Am: Food Anim Pract.

[B10] Stanek C, Frickh JJ, Karall P, Zemljic B (2004). Claw condition and meat quality factors in fattening bulls in two different housing systems. Proceedings of the 13th International Symposium and 5th Conference on Lameness in Ruminants: 11–15 February 2004; Maribor.

[B11] Lawrence RJ, Elliott R, Norton BW, Thoefner MB, Laxton I, Hueffner M, Zemljic B (2004). Influence of biotin supplementation on hoof chemical composition and rates of wear and growth in long fed F1 wague/black Angus Steers. Proceedings of the 13th International Symposium and 5th Conference on Lameness in Ruminants: 11–15 February 2004; Maribor.

[B12] Maton A, Wierenga HK, Peterse DJ (1987). The influence of the housing system on claw disorders with dairy cows. Cattle housing systems, lameness and behaviour.

[B13] Thysen I, Wierenga HK, Peterse DJ (1987). Foot and leg disorders in dairy cattle in different housing systems. Cattle housing systems, lameness and behaviour.

[B14] Faye B, Lescourret F (1989). Environmental factors associated with lameness in dairy cattle. Prev Vet Med.

[B15] Sogstad ÅM, Fjeldaas T, Østerås O, Forshell KP (2005). Prevalence of claw lesions in Norwegian dairy cattle housed in tie stalls and free stalls. Prev Vet Med.

[B16] Bergsten C (2001). Effects of conformation and hoof management system on hoof and leg diseases and lameness in dairy cows. Vet Clin North Am: Food Anim Pract.

[B17] http://www.ssb.no.

[B18] Bergsten C, Mortellaro CM, De Vecchis L, Brizzi A (2000). Workshop report about the documentation of claw diseases. Part 2. Proceedings of the 11th International Symposium on the Disorders of the Ruminant Digit: 3–7 September 2000; Parma.

[B19] Toussaint Raven E (1989). Cattle foot care and claw trimming.

[B20] De Boer H (1982). Animal production systems to meet the consumer demands – Western Europe. Proceedings of the International Symposium on Meat Science and Technology: Lincoln, Nebraska.

[B21] European Union Council Regulation (EEC) No 1026/91 of 22 April 1991 determining the Community scale for the classification of carcasses of adult bovine animals.

[B22] Manske T (2003). Om klövvårdares klövhälsoregisteringar. Sven Vet Tidn.

[B23] Sogstad ÅM, Østerås O, Fjeldaas T (2006). Bovine claw and limb disorders related to reproductive performance and production diseases. J Dairy Sci.

[B24] Manske T, Hultgren J, Bergsten C (2002). Prevalence and interrelationships of hoof lesions and lameness in Swedish dairy cows. Prev Vet Med.

[B25] Roeber DL, Smith GC, Floyd JG, Cowman GL, Mortellaro CM, De Vecchis L, Brizzi A (2000). Lameness in breeding cattle in The United States: The national market cow and bull beef quality audit, 1999. Proceeding of the 11th International Symposium on Disorders of the Ruminant Digit: 7–10 September 2000; Parma.

[B26] Hird DW, Weigler BJ, Salman MD, Danaye-Elmi C, Palmer CW, Holmes JC, Utterbach WW, Sischo WM (1991). Expenditures for veterinary services and other costs of disease and disease prevention in 57 California beef herds in the National Animal Health Monitoring System (1988–1989). J Am Vet Med Assoc.

[B27] Griffin D (1998). Feedlot diseases. Vet Clin North Am: Food Anim Pract.

[B28] Forshell KP, Fjeldaas T, Hjørungdal KM, Kleppa AL (2001). Claw disorders in a Norwegian dairy herd – a case study. Nor Vet Tidsskr.

[B29] Mülling C, Budras KD, Lischer CJ, Ossent P (1998). Influence of environmental factors on horn quality of the bovine hoof. Proceedings of the 10th International Symposium on Lameness in Ruminants: 7–10 September 1998; Lucerne.

[B30] Kujala M, Schnier C, Niemi J, Soveri T, Zemljic B (2004). Occurrence of hoof diseases in dairy cattle in Finland. Proceedings of the 13th International Symposium on Lameness in Ruminants: 11–15 February 2004; Maribor.

[B31] Somers JGCJ, Frankena K, Noordhuizen-Stassen EN, Metz JHM (2005). Risk factors for interdigital dermatitis and heel erosion in dairy cows kept in cubicle houses in the Netherlands. Prev Vet Med.

[B32] Bergsten C (1994). Haemorrhages of the sole horn of dairy cows as a retrospective indicator of laminitis: an epidemiological study. Acta Vet Scand.

[B33] Smilie RH, Hoblet KH, Eastridge ML, Weiss WP, Schnitkey GL, Moeschberger ML (1999). Subclinical laminitis in dairy cows: use of severity of hoof lesions to rank and evaluate herds. Vet Rec.

[B34] Greenough PR, Vermunt JJ, McKinnon JJ, Fathy FA, Berg PA, Cohen RDH (1990). Laminitis-like changes in the claws of feedlot cattle. Can Vet J.

[B35] Sogstad ÅM, Fjeldaas T, Østerås O (2005). Lameness and claw lesions of the Norwegian Red Dairy Cattle housed in free stalls in relation to environment, parity and stage of lactation. Acta Vet Scand.

[B36] Smits MCJ, Frankena K, Metz JHM, Noordhuizen JPTM (1992). Prevalence of digital disorders in zero-grazing dairy cows. Livest Prod Sci.

[B37] Smith DR, Brodersen BW (1998). Lesions of the hoof wall, sole, and skin associated with osteomyelitis of the distal third phalanx (toe abscess) and other secondary foot lesions in feedlot cattle. Conference for Research Workers in Animal Disease.

[B38] Mülling CKW, Shearer JK (2002). Theories on the pathogenesis of white line disease – an anatomical perspective. Proceedings of the 12th International Symposium on Lameness in Ruminants: 9–13 January 2002; Orlando.

[B39] David GP (1989). Epidemiological factors associated with a high incidence of sole ulcer and white line disease in dairy cattle. Proceedings of Society for Veterinary Epidemiology and Preventive Medicine: 12–14 April 1989; Exeter.

[B40] Fiedler A, Mortellaro CM, De Vecchis L, Brizzi A (2000). Comparative studies about the prevalence of claw diseases in tie-stalls and loose-housing systems in Bavaria 1998 and 1999 (Poster). Proceedings of the 11th International Symposium on Disorders of the Ruminant Digit: 3–7 September; Parma.

[B41] Clark CR, Petrie L, Waldner C, Wendell A (2004). Characteristics of the bovine claw associated with the presence of vertical fissures (sandcracks). Can Vet J.

[B42] Fjeldaas T, Sogstad ÅM, Østerås O (2006). Claw trimming routines in relation to claw lesions, claw shape and lameness in Norwegian dairy herds housed in tie stalls and free stalls. Prev Vet Med.

[B43] Fjeldaas T (1983). Klauv-og ekstremitetslidelser hos mjølkeku i relasjon til miljø. (Claw-and leg disorders in dairy cows in relation to the environment). MS thesis.

[B44] van Amstel SR, Palin FL, Shearer JK, Shearer JK (2002). Application of functional trimming procedures to corkscrew claws. Proceedings of the 12 th International Symposium on Lameness in Ruminants: 9–13 January 2002; Orlando.

[B45] Wells SJ, Trent AM, Marsh WE, Robinson RA (1993). Prevalence and severity of lameness in lactating dairy cows in a sample of Minnesota and Wisconsin herds. J Am Vet Med Assoc.

[B46] Ward WR (1999). Lameness in dairy cattle – an overview. Cattle Pract.

[B47] Offer JE, McNulty D, Logue DN (2000). Observations of lameness, hoof conformation and development of lesions in dairy cattle over four lactations. Vet Rec.

[B48] Townsend HGG, Meek AH, Lesnick TG, Janzen ED (1989). Factors associated with average daily gain, fever and lameness in beef bulls at the Saskatchewan central feed test station. Can J Vet Res.

[B49] Barth AD, Waldner CL (2002). Factors affecting breeding soundness classification of beef bulls examined at the Western College of Veterinary Medicine. Can Vet J.

[B50] Sogstad ÅM, Østerås O, Fjeldaas T (2007). Bovine claw and limb disorders related to culling and carcass characteristics. Livest Sci.

